# Supplementary material for: The interactome of intact mitochondria by cross-linking mass spectrometry provides evidence for coexisting respiratory supercomplexes
Source: Mol Cell Proteomics. 2017 Dec 8;17(2):216–32. doi: 10.1074/mcp.RA117.000470 (PMC5795388; doi:10.1074/mcp.RA117.000470)
Supplement: Supplemental Data [file supp_RA117.000470_133922_0_supp_23978_fzffwf.zip › spectra_annotation/mito_DR_spectra_annotation/185-1-3-1-22-1.pdf]

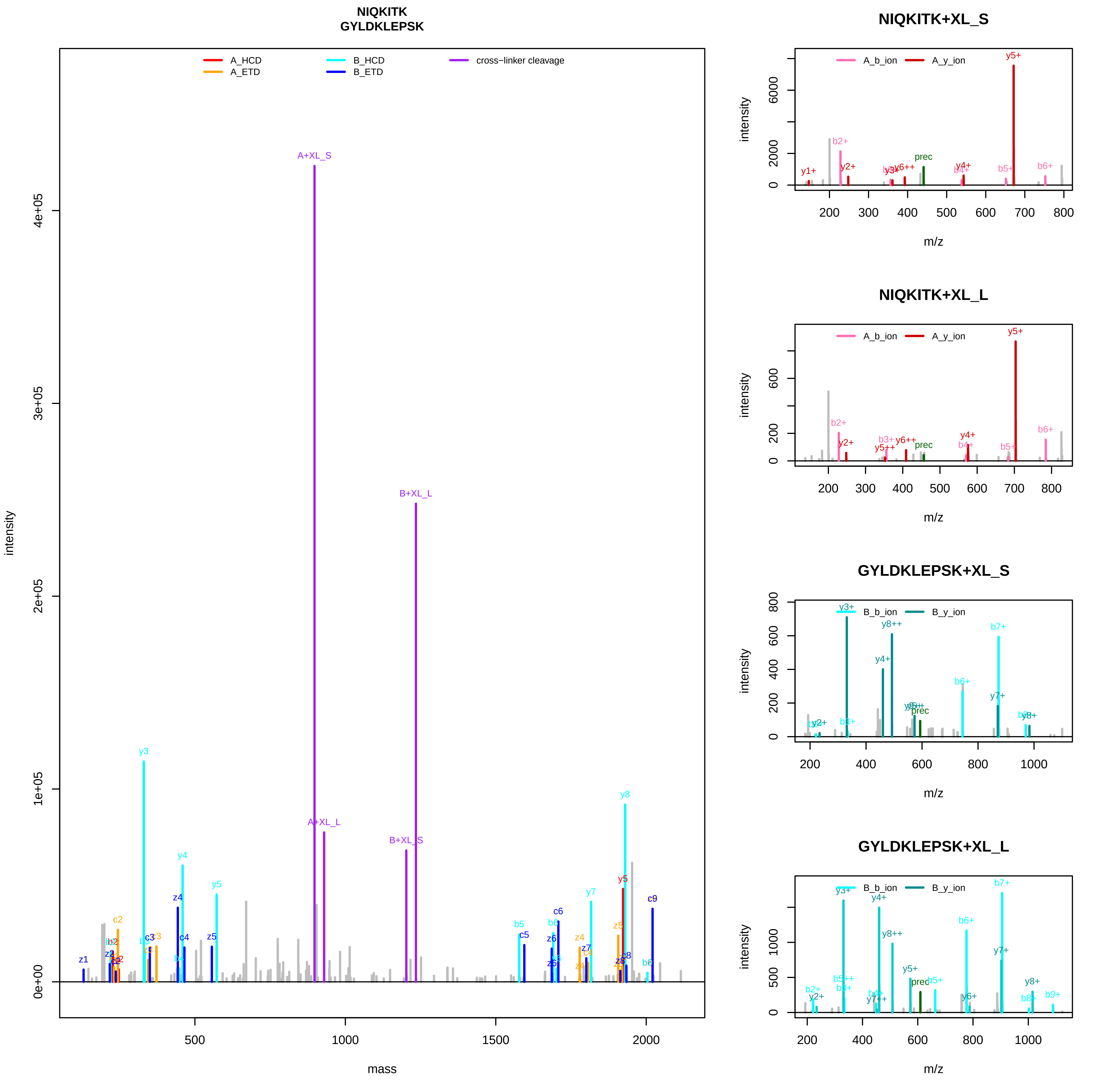
NIQKITK
GYLDKLEPSK
A_HCD
A_ETD
B_HCD
B_ETD
cross−linker cleavage
A+XL_S
B+XL_L
A+XL_L
B+XL_S
y3
y4
y5
y7
y8
z1
z4
z5
c4
c5
c6
c9
intensity
mass
0e+00
1e+05
2e+05
3e+05
4e+05
500
1000
1500
2000
NIQKITK+XL_S
NIQKITK+XL_L
GYLDKLEPSK+XL_S
GYLDKLEPSK+XL_L
A_b_ion
A_y_ion
B_b_ion
B_y_ion
prec
y5+
b2+
y2+
y4+
b6+
y3+
b7+
y8++
y4+
b6+
y7+
m/z
